# Probing depth is an independent risk factor for HbA1c levels in diabetic patients under physical training: a cross-sectional pilot-study

**DOI:** 10.1186/s12903-018-0491-9

**Published:** 2018-03-16

**Authors:** Katharina Wernicke, Sven Zeissler, Frank C. Mooren, Torsten Frech, Stephanie Hellmann, Meike Stiesch, Jasmin Grischke, Silvia Linnenweber, Bernhard Schmidt, Jan Menne, Anette Melk, Pascal Bauer, Andree Hillebrecht, Jörg Eberhard

**Affiliations:** 1 Department of Prosthetic Dentistry, Hannover Medical School, Hannover, Germany; 2 Faculty of Physical Education and Sports, Comenius University Bratislava, Bratislava, Slovakia; 3 Faculty of Sports Medicine, University of Giessen, Giessen, Germany; 4 Department of Nephrology, Hannover Medical School, Hannover, Germany; 5 Department of Pediatric Nephrology, Hannover Medical School, Hannover, Germany; 6 Clinic I, Cardiology and Angiology, University Hospital Giessen, Giessen, Germany; 7 Medical Department Volkswagen AG, Baunatal, Germany; 8 Faculty of Dentistry, Charles Perkins Centre, Westmead Centre for Oral Health, University of Sydney, Sydney, New South Wales 2145 Australia

**Keywords:** Periodontitis, Diabetes mellitus type 2, Physical training, Lifestyle intervention, Oral health

## Abstract

**Background:**

This cross-sectional study investigates the potential association between active periodontal disease and high HbA1c levels in type-2-diabetes mellitus subjects under physical training.

**Methods:**

Women and men with a diagnosis of non-insulin-dependent diabetes mellitus and ongoing physical and an ongoing exercise program were included. Periodontal conditions were assessed according to the CDC-AAP case definitions. Venous blood samples were collected for the quantitative analysis of HbA1c. Associations between the variables were examined with univariate and multivariate regression models.

**Results:**

Forty-four subjects with a mean age of 63.4 ± 7.0 years were examined. Twenty-nine subjects had no periodontitis, 11 had a moderate and 4 had a severe form of periodontal disease. High fasting serum glucose (*p* < 0.0001), high BMI scores (*p* = 0.001), low diastolic blood pressure (*p* = 0.030) and high probing depth (*p* = 0.036) were significantly associated with high HbA1c levels.

**Conclusions:**

Within the limitations of this study HbA1c levels are positively associated with high probing pocket depth in patients with non-insulin-dependent diabetes mellitus under physical exercise training. Control and management of active periodontal diseases in non-insulin-dependent patients with diabetes mellitus is reasonable in order to maximize therapeutic outcome of lifestyle interventions.

## Background

Type 2 diabetes mellitus (T2DM) is the most common chronic metabolic disease in the Western World and it is characterized by high blood glucose levels. In 1970 it was observed for the first time that patients with T2DM had a high risk for periodontal disease [1]. During the following decades several epidemiological and interventional studies documented a link between T2DM and periodontitis and several systematic reviews and meta-analysis confirmed this association [2, 3]. It was shown that the risk of periodontitis was 3 to 4 times higher in diabetic patients compared to systematically healthy individuals [4]. Furthermore, a two-way relationship was postulated among T2DM and periodontitis [5]. However, an interventional study could not demonstrate a significant reduction of HbA1c levels following non-surgical periodontal therapy in patients suffering from moderate to advanced chronic periodontitis [6].

Potential mechanistic links between diabetes and chronic periodontitis include the local and systemic inflammatory host response, the presence of advanced glycation endproducts (AGE), oxidative stress and mitochondrial dysfunction [3, 7]. It is hypothesized that periodontal inflammation or pathogenic bacteria and their by-products induce the synthesis of acute phase proteins, cytokines and oxidative stress molecules in the liver that subsequently reduce insulin sensitivity [5].

Lifestyle and health interventions like physical activity are capable to modulate this relationship between periodontal diseases and T2DM. Generally, physical activity improves the metabolic state of T2DM patients, decreases HbA1c levels and has a positive effect on cardiovascular mortality, quality of life, lipids and blood pressure [8, 9]. Physical activity on the other hand is significantly associated with a low prevalence of periodontitis [10, 11] and subjects with high levels of physical activity had significantly lower plaque indices, gingival indices and clinical attachment loss compared to sedentary subjects [12]. Reactive oxygen species (ROS) are potentially seen to be a molecular link between physical activity, T2DM and periodontitis. Systemic ROS levels were down-regulated by physical exercise, whereas chronic periodontitis had the opposite effect [13, 14]. Furthermore, high systemic ROS levels declined the glycaemic control [15].

To further investigate the associations between T2DM, periodontitis and physical exercise and to test the hypothesis that poor periodontal conditions are a risk factor for the decline of positive effects of physical exercise on glycaemic control the following cross-sectional study was designed.

## Methods

### Study population

The study population was recruited from a German fitness centre by advertisement over a period of 6 months (8/2011 to 1/2012). Informed consent was obtained from all participants included in the study. A comprehensive medical history was recorded. The inclusion criteria were: Women and men aged between 18 and 79 years with a diagnosis of a non-insulin-dependent T2DM and an ongoing physical exercise program of 6 months duration. Exclusion criteria were: unstable coronary artery disease, inadequately controlled arterial hypertension, severe orthopaedic limitations, advanced diabetic retinopathy and diabetic foot syndrome. This cross-sectional trial was approved by the local Ethics Committee of the Justus-Liebig University Giessen, Germany (reference number 2011–0006).

### Physical training intervention

The training intervention of the participants was either an equipment-supported resistance-training group (Milon endurance circle) an aerobic training group (cardio equipment) or a combination of both [9]. The training was standardized and intensively supervised. The range was extended at least up to 90 min per week and was automatically controlled by electronic recordings and monitored by experienced personal.

### Periodontal examination

A professional dentist recorded probing depth (PD), bleeding on probing (BOP), plaque-index (PBI), suppuration and dental findings. The periodontal measurements were done using a pressure calibrated periodontal probe (Florida Probe-System) at 6 sides per tooth, BOP and plaque indices were recorded at 4 sides per tooth. The existence of “no or mild”, “moderate or severe” periodontal conditions were determined by the clinical case definitions of periodontitis introduced by the Centres for Disease Control and Prevention and the American Academy of Periodontology (CDC-AAP) [16]. “No or mild” periodontitis was stated if neither moderate nor severe periodontitis was determined. Patients were categorized as moderate periodontitis by ≥2 inter proximal sides with CAL ≥4 mm, or by ≥2 inter proximal sides with PD ≥5 mm (not at adjacent teeth).

### Laboratory assessment

Venous blood samples were collected after overnight fasting. Measurements included serum glucose, cholesterol, LDL, HDL, and hsCRP using the Modular P800 analytical device (Roche Institute). For the quantitative analysis of HbA1c an Adams A1c HA8180V analysis machine was used (Axon Lab AG, Reichenbach, Germany**).** A urine test was used to determine microalbumine.

### Oral glucose tolerance test

Oral glucose tolerance test was performed with 75 g glucose solution in the morning after overnight fasting. After 60 and 120 min serum glucose and C-peptide levels were measured.

### Blood pressure and analysis of oxygen consumption

Systolic and diastolic blood pressures were measured with a stethoscope and a blood pressure cuff according to the WHO guidelines. Blood pressure was measured after a 5 min rest period before starting the exercise by a trained physician under standardised conditions. A second measurement was done 10 min after the initial measurement and the mean blood pressure was calculated. Spiroergometry (bicycle ergometer) as cardiopulmonary exercise test was used to identify VO_2peak_.

### Statistical analyses

Statistical analysis was conducted in the statistical software package SPSS 19.0 (IBM Corp., Armonk, NY, USA). Descriptive statistics integrated calculation of mean values and standard deviations for quantitative variables. Frequency and percentage were expressed as qualitative variables. Differences among the HbA1c levels were analysed using ANOVA for continuous variables and the chi-square or Fischer’s exact test, each for categorical variables. The significance level was defined as α = 0.05.

The association between the variables was examined with univariate regression models, with HbA1c levels as the dependent variable. All variables with a *p*-value *p* < 0.2 were considered significant and were included in a multivariate regression model. Correlations among variables were checked with Pearson correlation coefficient and extremely dependent variables were excluded from analysis. A multivariate analysis (backward stepwise linear regression with *p* = 0.10 to enter and *p* = 0.05 to exit) was performed. Feasible predictors were sex, age, BMI, physical activity, VO_2max_, heart rate, diastolic blood pressure, cholesterol, HDL, LDL, hsCRP, glucose serum (fasting), glucose serum (after 2 h), bleeding on probing, pocket depth and periodontal disease severity (no/mild, moderate or severe periodontitis).

## Results

One-hundred-ten subjects met the inclusion criteria and forty-four Caucasian subjects (mean age 63.4 ± 7.0 years) signed the informed consent to participate in the dental assessment. Twenty-nine subjects had no periodontitis, eleven had a moderate and four had a severe form of periodontal disease. Descriptive statistics for clinical case definitions and HbA1c levels are shown in Table 1. The differences between HbA1c levels in patients with no or mild, moderate or severe periodontitis were statistically not significant.

**Table 1:** HbAlc serum concentration did not significantly increased with periodontal disease severity (*p* = 0.977)

		HbAlc (mmol/mol) mean ± SD 95% Cl
CDC-AAP	no/mild	51.7 ± 5.1 [43.5; 59.8]
	moderate	50.3 ± 11.1 [42.9; 57.8]
	severe	50.6 ± 10.9 [46.4; 54.7]

Individuals with HbA1c serum concentrations in the lower quartile indicated lower weight (*p* = 0.016) and BMI scores (*p* = 0.003), high levels of VO_2peak_ (*p* = 0.008), low systolic blood pressure (*p* = 0.031), low concentrations of fasting glucose (*p* < 0.0001) as well as low levels of glucose 120 min after glucose tolerance test (*p* < 0.0001), low levels of serum hsCRP (*p* = 0.006), low bleeding on probing (*p* = 0.023) and low plaque indices (*p* = 0.031) (Table 2). No variations were found between different HbA1c quartiles for age, HR, cholesterol, LDL, HDL, pocket depth and periodontitis disease severity or physical activity.

**Table 2:** Descriptive statistics (mean ± standard deviation) for participating subjects and ANOVA analysis for quartiles of HbAlc (mmol/mol)

	All participants (*n* = 44)	HbA1c (mmol/mol)				
		1. Quartile (*n* = 13)	2. Quartile (*n* = 6)	3. Quartile (n = l5)	4. Quartile (*n* = 10)	*P*
Age, y	63.4 ± 7.0	65.2 ± 5.6	63.7 ± 9.8	63.5 *±* 5.8	60.8 ± 8.8	0.713
Weight, kg	91.6 ± 20.1	81.8 ± 17.9	81.7 ± 15.0	95.1 ± 18.9	105.3 ± 19.9	0.016
BM1	32.1 ± 6.7	28.6 ± 4.9	27.4 ± 2.4	34.0 ± 7.1	36.7 ± 6.0	0.003
VO2_peak,_ mL/mm/kg	23.0 ± 6.0	23.8 ± 5.1	27.3 ± 4.8	24.2 ± 6.3	18.0 ± 4.4	0.008
Heart rate, bpm	70.4 ± 11.0	71.6 ± 12.0	71.5 ± 9.0	67.9 ± 10.1	72.1 ± 12.7	0.403
Systolic blood pressure, mm Hg	129.7 ± 19.9	121.4 ± 9.9	117.3 ± 8.7	139.1 ± 27.4	133.9 ± 13.5	0.032
Diastolic blood pressure, mm Hg	82.3 ± 13.0	79.0 ± 9.2	74.3 ± 7.0	87.4 ± 16.2	83.8 ± 12.3	0.132
Serum glucose (fasting), mmol/l	7.8 ± 1.9	6.5 ± 1.6	7.2 ± 0.8	7.5 ± 1.1	10.2 ± 1.2	< 0.0001
Semm glucose (2 h), mmol/L	12.1 ± 4.6	9.4 ± 4.1	12.8 *±* 2.5	10.8 ± 4.0	17.1 ± 2.6	< 0.0001
Total cholesterol, mmol/L	4.9 ± 1.2	4.9 ± 1.3	4.3 ± 0.6	4.9 ± 1.4	5.1 ± 0.9	0.588
LDL cholesterol, mmol/L	3.1 ± 1.1	3.0 ± 1.1	2.5 *±* 0.9	3.2 ± 1.4	3.3 ± 0.7	0.539
HDL cholesterol, mmol/L	1.5 ± 0.4	1.6 ± 0.4	1.4 *±* 0.4	1.4 ± 0.3	1.3 ± 0.4	0.133
hsCRP, mg/L	1113.6 ± 1783.6	331.6 ± 312.6	858.8 ± 1838	.4744.9 ± 537.0	2853.5 2935.6	0.006
Bleeding (BOP), %	5.0 ± 8.1	3.9 ± 5.3	1.1 ± 1.9	3.2 ± 4.7	11.6 ± 13.2	0.023
Plaque Index (PI), %	49 ± 38.4	39.6 ± 36.3	24.0 ± 26.2	48.8 ± 38.5	76.6 ± 35.1	0.031
Pocket depth, mm	1.3 ± 0.3	1.3 ± 0.2	1.2 ± 0.3	1.3 ± 0.3	1.4 ± 0.2	0.354

*LDL* low-density lipoprotein, *HDL* high-density lipoprotein *hsCRP* high-sensitive C-reactive protein

The univariate regression analysis demonstrated that heart rate (*p* = 0.016), fasting serum glucose (*p* < 0.0001), cholesterol (*p* = 0.031), LDL (0.034), hsCRP (*p* < 0.0001) and bleeding on probing (*p* < 0.0001) were significantly associated with HbA1c serum concentrations (Table 3). Variables kept in the multivariate regression analysis after the stepwise selection procedures were fasting serum glucose, VO_2peak_, diastolic blood pressure, probing depth and BMI. High fasting serum glucose (*p* < 0.0001), low diastolic blood pressure (*p* = 0.030), high probing depth (*p* = 0.036) and high BMI scores (*p* = 0.001), were significantly associated with high HbA1c levels.

**Table 3:** Uni- and multivariate regression analysis with HbAlc (mmol/mol) as the dependent variable. The regression coefficient corresponds to a one-unit change of the dependent variable

	Univariate regression		
	Regression coefficient	95% CI	*P*-value
Age, y	1.333		0.264
Sex	0.806		0.695
BMI	1.634		0.139
VO_*2*peak,_ mL/min/kg	1.200		0.350
Heart rate, bpm	2.682		0.016
Systolic blood pressure, mm Hg	0.403		0.982
Diastolic blood pressure, mm Hg	1.467		0.982
Serum glucose (fasting), mmol/L	6.194		< 0.0001
Serum glucose (2 h) mmol/L	1.412		0.223
Total cholesterol, mg/dL	2.339		0.031
LDL cholesterol, mmol/L	2.295		0.034
HDL cholesterol, mmol/L	0.905		0.597
hsCRP, mg/L	6.725		< 0.0001
Plaque Index (PI)	1.651		0.134
Probing depth, mm	0.888		0.614
Bleeding (BOP), %	6.21		< 0.0001
Clinical Case Definition, CDC-AAP			
No or mild Periodontitis	0.299		0.997
Moderate Periodontitis	0.950		0.553
Severe Periodontitis	1.367		0.245
	Multivariate regression		
	Regression	95% CI	*P*-value
	coefficient		
Serum glucose (fasting), mmol/L	3.666	2.239; 5.093	< 0.0001
Diastolic blood pressure, mm Hg	−0.242	−0.459; −0.025	0.030
Probing depth, mm	6.845	0.480; 13.210	0.036
BMI	0.796	0.330; 1.262	0.001

*CI* confidence interval

## Discussion

This is the first study that identified probing depth, a clinical marker of active periodontal disease [16], as a risk factor for a poor glycaemic status of subjects under physical exercise training that was aimed to reduce HbA1c levels. In addition to studies showing that periodontal disease increased the risk for systemic diseases, the results of the present study demonstrate that poor periodontal conditions are an independent risk factor for the decline of positive outcomes of health care interventions. In accordance with the present findings, it was recently shown that poor periodontal health attenuated the positive effects of physical exercise for markers of biological aging [17]. For T2DM patients life style interventions including physical exercise or dietary counselling are of critical importance to reduce HbA1c levels [9]. However, chronic inflammatory processes such as periodontal disease may negatively affect the overall outcome of these lifestyle interventions.

This study showed an association between periodontal probing depth and the HbA1c levels in women and men with T2DM under physical exercise training; stepwise regression analysis showed that fasting glucose levels, cardiorespiratory fitness, diastolic blood pressure, probing depth and BMI are risk factors for high concentrations of HbA1c in this population. The selected population showed several risk factors for high HbA1c levels and benefit from the non-insulin dependent reduction of HbA1c levels and lifestyle interventions. Obesity is one of the main factors in the pathogenesis of T2DM, accordingly, the study participants were overweight or obese with respect to BMI scores between 27 and 36. Overweight and obese subjects are less able to process insulin effectively and therefore the observed association between BMI and high HbA1c concentrations was in accordance with previous findings [18]. In addition to established risk factors probing depth was identified as a risk factor for high HbA1c levels as well, which is in accordance to the repeatedly shown two-way association between periodontitis and T2DM. The clinical case definition according to CDC-AAP criteria, plaque indices and bleeding frequency were also recorded to characterize periodontal health, but were not identified as risk factors in the following statistical analysis. Only bleeding on probing was significantly associated with HbA1c levels in the univariate regression analysis. If such a relationship indeed exists further research with other study designs would be necessary to provide conclusions related to causality.

The mechanisms that link diabetes and periodontitis are not completely understood, but involve aspects of inflammation and immune function [6]. T2DM is associated with higher serum levels of inflammatory mediators such as interleukin-1β and tumor necrosis factor-α [19] and the local overproduction of these cytokines is a major contributor to alveolar bone destruction in periodontitis [20]. The presence of AGE in the gingival tissues, resulting in the up-regulation of cytokine secretion and the generation of ROS contribute to the local tissue damage in diabetic patients as well [21]. In contrast, physical exercise is capable to reduce the accumulation of ROS and hence positively affects the systemic inflammatory burden [22]. Indeed, recently moderate and severe periodontitis were identified as independent risk factors for low cardiorespiratory fitness [11]. Physical exercise reduces risks for high HbA1c levels, overweight [23, 24] and high C-reactive protein concentrations in healthy adults [25]. This discussion led to the conclusion that inflammation is the common link between physical activity, metabolic and periodontal diseases.

In conclusion the present study showed that HbA1c levels are positively associated with high probing pocket depth measurements in the present population. For external validity it will be of value to address other physical exercise programs and lifestyle interventions in the selected population and in insulin-dependent patients as well. From these data it might be reasonable to control and manage periodontal disease in non-insulin-dependent patients in order to maximize the therapeutic outcome of lifestyle interventions. However, the next steps are interventional studies, to address a potential causal relationship.

## Conclusions

The present study showed that high HbA1c levels are positively associated with clinical parameters of periodontitis in subjects suffering of non-insulin dependent diabetes under a physical exercise program. Subject to additional evidence, it will be beneficial to prevent and treat periodontal disease in non-insulin-dependent patients in order to maximize the therapeutic outcomes of lifestyle interventions.

## Abbreviations

AGE: Advanced glycation end products
ANOVA: Analysis of variance
BMI: Body Mass Index
BOP: Bleeding on probing
CAL: Clinical attachment level
CDC-AAP: Centre of disease control - American academy of periodontology
HbA1c: Glycated haemoglobin
HDL: High-density lipoprotein
HR: Heart rate
hsCRP: High-sensitivity C-reactive protein
LDL: Low-density lipoprotein
PBI: Plaque Index
PD: Probing depth
ROS: Reactive oxygen species
T2DM: Type 2 diabetes mellitus
VO_2peak_: Peak oxygen uptake
